# Real-world experiences with brentuximab vedotion-based regimens in systemic anaplastic large cell lymphoma: a multi-center retrospective study

**DOI:** 10.3389/fonc.2024.1494384

**Published:** 2025-01-07

**Authors:** Zhiqiang Zhao, Qinchuan Yu, Liping Su, Jianxia He, Jie Tao, Yanfeng Xi, Yujiao Guo, Yanhong Luo, Lieyang Wang

**Affiliations:** ^1^ Department of Hematology, Shanxi Province Cancer Hospital/ Shanxi Hospital Affiliated to Cancer Hospital, Chinese Academy of Medical Sciences/Cancer Hospital Affiliated to Shanxi Medical University, Taiyuan, Shanxi, China; ^2^ Department of Hematology, Shanxi Provincial People’s Hospital, Taiyuan, Shanxi, China; ^3^ Department of Hematology, First Hospital of Shanxi Medical University, Taiyuan, Shanxi, China; ^4^ Department of Health Statistics, Shanxi Medical University, Taiyuan, Shanxi, China

**Keywords:** real world study, lymphoma, china, brentuximab vedodin, systemic anaplastic large cell lymphoma(sALCL)

## Abstract

**Background:**

Brentuximab vedotin (BV) has demonstrated high remission rates in clinical trials for systemic anaplastic large cell lymphoma (sALCL), yet its real-world effectiveness in China remains unconfirmed. This retrospective observational study evaluates BV-based regimens in patients with sALCL, treated from 2020 to 2023.

**Methods:**

A multi-center observational retrospective study was conducted on patients with sALCL received BV plus cyclophosphamide, doxorubicin, and prednisone (CHP) upfront or BV plus gemcitabine, oxaliplatin(GemOx), gemcitabine, cisplatin, dexamethasone(GDP), or isocyclophosphamide, carboplatin, etoposide (ICE)for later lines. Primary endpoints were complete response rate (CRR) and overall response rate (ORR); secondary endpoints included progression-free survival (PFS), overall survival (OS), duration of response (DOR), and the incidence of adverse events (AEs).

**Results:**

Among the 38 patients (28 newly diagnosed and 10 with refractory/relapsed disease), the ORR were 100% (with 89.3% CR) for newly diagnosed patients and 70% (with 50% CR) for refractory/relapsed patients. The median duration of response was 14 months for newly diagnosed patients and 23.8 months for those with refractory/relapsed disease. 2-year Survival rates were 100% for newly diagnosed patients and 80% for refractory/relapsed patients, with 2-year PFS rates of 92.8% and 70%, respectively. Neurological toxicities were commonly observed but resolved following the completion of treatment.

**Conclusion:**

BV has proven to be effective and well-tolerated in real-world settings for the treatment of sALCL, reinforcing its potential as a promising option for first-line or subsequent therapy. The sustained efficacy observed post-CR suggests that these patients may have a prolonged disease control.

## 1 Introduction

sALCL is a subtype of peripheral T-cell lymphoma, and includes cases with and without translocations of the anaplastic lymphoma kinase gene (ALK). CD30 is a key molecule that is involved in ALCL pathogenesis, diagnosis, and treatment (1). Several therapeutic agents have been developed for sALCL, but Brentuximab vedotin (BV), an innovative antibody-drug conjugate that specifically targets CD30+ malignant cells, marks a significant recent advancement (2, 3). BV has shown high ORR in both newly diagnosed and refractory/relapsed sALCL cases in clinical trials (4–6). However, despite these promising results in controlled clinical settings, the real-world effectiveness and safety profile of BV among sALCL patients in China remain underexplored. Since its May 2020 approval from the National Medical Products Administration (NMPA) and subsequent introduction into the Chinese market (7), there has been a pressing need to evaluate if its clinical trial outcomes will translate into routine clinical practice. This knowledge gap underscores the importance of conducting comprehensive observational studies to assess BV’s real-world applications and efficacy.

To address this need, we conducted a retrospective observational study focused on BV-based treatments for sALCL within the context of everyday clinical practice in China. We aimed to verify and replicate the clinical trial findings in a real-world setting, and provide valuable insights into BV’s efficacy, safety, and comprehensive treatment outcomes for sALCL. Overall, we hope to contribute to optimizing therapeutic strategies for sALCL, and ensure that patients receive the most effective and safe treatments based on both clinical evidence and real-world experience.

## 2 Methods

### 2.1 Study design and population

The retrospective observational multi-center study was conducted among patients with sALCL who were treated with either BV plus CHP (for newly diagnosed cases) or BV-based combination regimens(GemOx, GDP or ICE) (for second-line and subsequent treatments) between September 2020 and October 2023. Completion of at least 4 cycles of BV combined therapy was required for inclusion in the study. The data was collected from three hospitals, including Shanxi Province Cancer Hospital,Shanxi Provincial People’s Hospital and the First Hospital of Shanxi Medical University. The study design was approved by the ethical committee in all participating hospitals and ensured compliance with the ethical standards delineated in the 1964 Declaration of Helsinki and its subsequent amendments. We obtained special permission from our ethical committee to collect data regarding patients who could not consent because they had died or been lost to follow up.

Prior to treatment, all patients had to have received a pathologically-confirmed diagnosis of sALCL, and underwent comprehensive pre-treatment assessments, including physical exams, routine hematology and biochemistry testing, and whole-body positron emission tomography (PET)/computed tomography (CT) imaging.

Newly diagnosed sALCL(ND-sALCL) patients were administered BV (1.8 mg/kg d1) in combination with CHP(cyclophosphamide 750 mg/m^2^ d1, doxorubicin 40-50 mg/m^2^ d1,Prednisone 100 mg d1-5)every three weeks. Refractory/Relapsed sALCL(R/R-sALCL) patients were administered BV (1.8 mg/kg d1) in combination with either GemOx(gemcitabine 1000 mg/m^2^ d1,d8, oxaliplatin 100 mg/m^2^ d1), GDP(gemcitabine 1000 mg/m^2^ d1,d8, cisplatin 75 mg/m^2^ d1, dexamethasone 40mg d1-4), or ICE(isocyclophosphamide 5g/m^2^ d2 equivalent dose of mesna relief, carboplatin(Based on AUC=5, the single dose ≤ 800mg)d2, etoposide 100mg/m^2^ d1-3). Treatment involved a 30-minute infusion of BV at a dose of 1.8 mg/kg of body weight every three weeks for up to a maximum of eight cycles.

Patients who did not meet these criteria were excluded from the study. The study’s primary endpoints were CRR and ORR achieved during the treatment with BV, and secondary endpoints included PFS, OS, DOR and AEs.

Response assessments were conducted using whole-body PET or CT scans with contrast following treatment cycles 2, 4, 6, and 8, and then every three months within the first year after discontinuation of the drug, and then every six months after that, based on the International Working Group revised response criteria for malignant lymphoma (8). Safety evaluations were used to record the incidence, severity, and type of any adverse events, and were based on the National Cancer Institute Common Terminology Criteria for Adverse Events (version 4.0).

### 2.2 Statistical analysis

The study utilized SPSS 25.0 for data analysis. Normally distributed measurements were presented as () and assessed using t-test. Categorical data were expressed as *n*( $\bar{\text{X}}\pm S$ ) and compared by means of the χ2 test. Additionally, in this research, we employed R to create separate swimmer plots that reflect the treatment responses of 28 ND-sALCL patients and 10 R/R-sALCL patients treated with BV. Furthermore, Kaplan-Meier survival analyses were conducted for a collective group of 38 patients to evaluate progression-free survival (PFS) and overall survival (OS). The log-rank test was applied to discern any significant differences between the survival curves. The Reverse Kaplan-Meier method is used to calculate the median follow-up time and its 95% confidence interval.

## 3 Study results

### 3.1 Study patients

A total of 41 patients were enrolled in the study. Among them, two newly diagnosed patients and one relapsed patient discontinued treatment with BV after one and two cycles, respectively, due to financial constraints. These three patients were excluded from the efficacy analysis as they did not complete the required four cycles of treatment; however, they were included in the safety analysis. Consequently, a total of 38 patients were treated with BV-based combination regimens, including 28 with ND-sALCL and 10 with R/R-sALCL. All patients had histologically confirmed sALCL.

The ND-sALCL group was comprised of 18 males (64.3%) and 10 females (35.7%), with a median age of 40 years (range: 13–70 years). Among these patients, 13 (46.4%) were ALK-positive and 15 (53.6%) wereALK-negative. A majority of patients (23; 82.1%), were diagnosed with stage III/IV disease. Bulky disease was identified in three patients (10.7%), and bone marrow involvement was noted in five patients (17.9%). B symptoms were observed in 16 patients (57.1%), and extranodal involvement was found in 10 patients (35.7%). Sequential autologous stem cell transplantation (ASCT) was performed in five cases (17.9%), and two patients (7.1%) received sequential radiotherapy. (Patient demographics and baseline characteristics are summarized in **Table 1**).

**Table 1:** Patient demographics and baseline characteristics.

Characteristic	ND-sALCL	R/R-sALCL
Total population, N	28	10
ALK-positive,n (%)	13(46.4)	5(50.0)
ALK-negative,n (%)	15(53.6)	5(50.0)
Median age, years (range)	40(13-70)	45.5(21-78)
Median time from BV, months (range)	15.3(4.1-32.2)	8.3(5.1-36.0)
Male, n (%)	18 (64.3)	6 (60.0)
Stage, n (%)		
I/II	5 (17.9)	0 (0)
III	7 (25.0)	3 (30.0)
IV	16 (57.1)	7 (70.0)
Bulky disease, n (%)	3 (10.7)	3 (30.0)
B symptoms, n(%)	16 (57.1)	6 (60.0)
IPI≥3, n(%)	8 (28.6)	9 (90.0)
ECOG>2,n(%)	9 (32.1)	6 (60.0)
Extranodal sites>1, n (%)	10 (35.7)	6 (60.0)
Bone marrow involvement, n (%)	5 (17.9)	4 (40.0)
Median number of previous therapies (range)	0 (0)	3 (1-5)
Sequential autologous stem cell transplant, n (%)	5 (17.9)	3 (30.0)
Sequential radiotherapy, n (%)	2 (7.1)	1 (10.0)
Retreatment with BV, n (%)	0	1 (10.0)

Median time from BV: the median time from the start of BV treatment to the data cutoff point.

The R/R-sALCL subgroup included six males (60%) and four females (40%), with a median age of 45.5 years (range: 21–78 years). This group was evenly split between ALK-positive and ALK-negative patients, with each accounting for 50%. The median time from diagnosis to initiation of BV therapy (or retreatment) was 29.8 months. Stage III disease was present in three patients (30%), and seven patients (70%) had stage IV disease. Bulky disease was found in three patients (30%), and four patients (40%) had bone marrow involvement. B symptoms were present in 60% of cases. The median number of prior treatments was three (range: 1–5), including two patients (20%) who previously received ASCT, and three patients (30%) who had previously received radiotherapy. Out of the 10 R/R patients in our study, 4 chose the ICE regimen, 4 opted for the GDP scheme, and 2 selected the GeMOX scheme. None of the patients received ALK inhibitors before. The detailed treatment history of R/R patients are outlined in **Supplementary Table S1**. Following remission, three patients (30%) underwent ASCT, and one patient (10%) received local radiotherapy. One patient (10%) who had previously been treated with BV combined with CHP as a first-line regimen relapsed nine months after discontinuing treatment and receiving retreatment with BV-ICE. (Patient demographics and baseline characteristics are summarized in **Table 1**).

### 3.2 Response to treatment

All patients completed a minimum of four cycles of the BV combination regimens, and were administered a median of 6.5 cycles (ranging from 4 to 8 cycles).

For ND-sALCL patients, the best ORR was 100% (25 CRs and 3 PRs), which slightly decreased to 92.9% by the EOF(end of follow-up) (25 CRs and 1 PR) (**Table 2**). All patients who achieved CR maintained their responses. However, two patients with PR eventually developed progressive disease (PD) while receiving BV therapy and were shifted to second-line treatments. Subsequently, one of these patients underwent sequential autologous stem cell transplantation and ultimately achieved CR, and another received an experimental therapy. Five ALK-negative patients who reached CR received consolidative autologous hematopoietic stem cell transplantations. The remaining nine ALK-negative patients with CR who did not undergo stem cell transplantation and did not have any disease progression at the time of last follow-up. Two patients who presented with initial masses underwent local radiotherapy after BV treatment. The end of follow-up ORR for ALK-positive ND-sALCL patients was 92.3% and 93.3% for ALK-negative ND-sALCL patients, with no significant differences between the two groups.

**Table 2:** Overall treatment responses and durability.

Results	ND-sALCL(28 cases)	R/R-sALCL(10 cases)
Best response		
CR, n (%)	25(89.3%)	5(50.0%)
PR, n (%)	3(10.7%)	4(40.0%)
SD, n (%)	0(0)	0(0)
PD, n (%)	0(0)	1(10.0%)
ORR, n (%)	28(100%)	9(90%)
Response at EOF		
CR, n (%)	25(89.3%)	5(50.0%)
PR, n (%)	1(3.6%)	2(20.0%)
SD, n (%)	0(0)	0(0)
PD, n (%)	2(7.1%)	3(30.0%)
ORR, n (%)	26(92.9%)	7(70.0%)
Median duration of response (CR) months (range)	14.0(2.1-27.6)	23.8(3.2-33.1)
Median PFS (months)	NR	NR
Median OS (months)	NR	NR

ND-sALCL, newly diagnosed sALCL; R/R-sALCL, refractory/relapsed sALCL; CR, complete response; PR, partial response; SD, stable disease; PD, progressive disease; ORR, overall response rate; EOF, end of follow-up; PFS, progression-free survival; OS, overall survival; NR, not reached.

In the R/R-sALCL group, the best ORR was 90% (5 CRs and 4 PRs), but this was reduced to 70% by the EOF (5 CRs and 2 PRs) (**Table 2**). All patients with CR maintained their response status. Of the patients who had PR initially, two progressed to PD during subsequent BV treatments. One of these patients received experimental therapy and is still alive, while the other suffered from bone marrow invasion and a large neck mass, and eventually died while receiving palliative radiotherapy. The tumor burden in the other two PR patients(one of the patients had undergone ASCT at first-line) decreased following additional BV combination therapy, but they did not achieve CR eventually, and entered other clinical trials later. Of the five CR patients, three underwent consolidative autologous hematopoietic stem cell transplantations, and one patient with an initial mass received local radiotherapy. Of the 5 ALK (+) patients, 4 achieved CR, while of the 5 ALK (-) patients, only 1 achieved CR(the patient underwent ASCT at first-line) and 2 achieved PR.

### 3.3 Long-term outcomes

All patients were followed for a median duration of 13.7 months (95% CI:12.5-18.3)(ranging from 4.1 to 36.0 months) after receiving their first dose of BV. For patients who achieved CR, the estimated median response duration was 14.0 months (ranging from 2.1 to 27.6 months) for ND-sALCL and 23.8 months (ranging from 3.2 to 33.1 months) for R/R-sALCL (**Table 2**).

**Figure 1:**
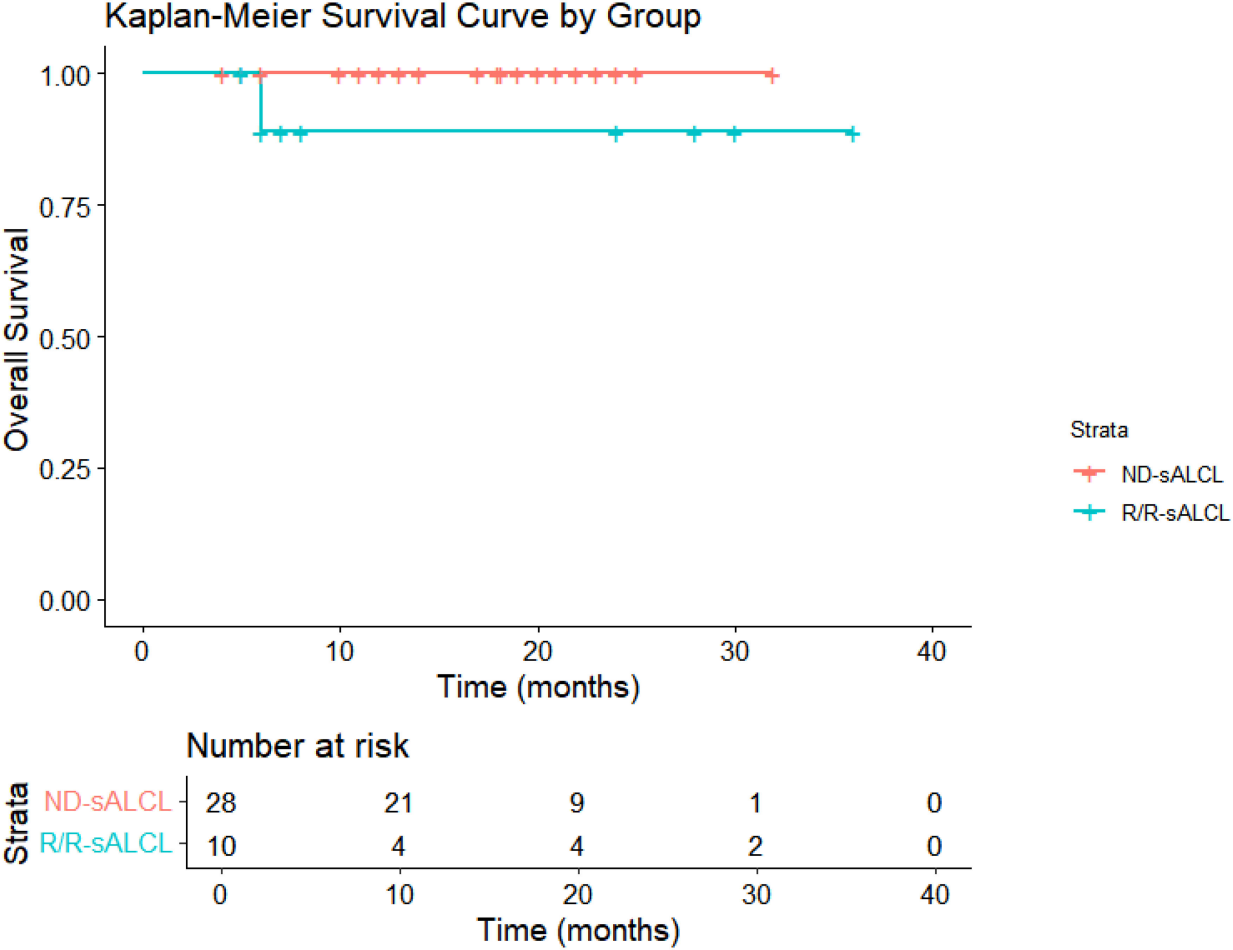
Overall survival.

**Figure 2:**
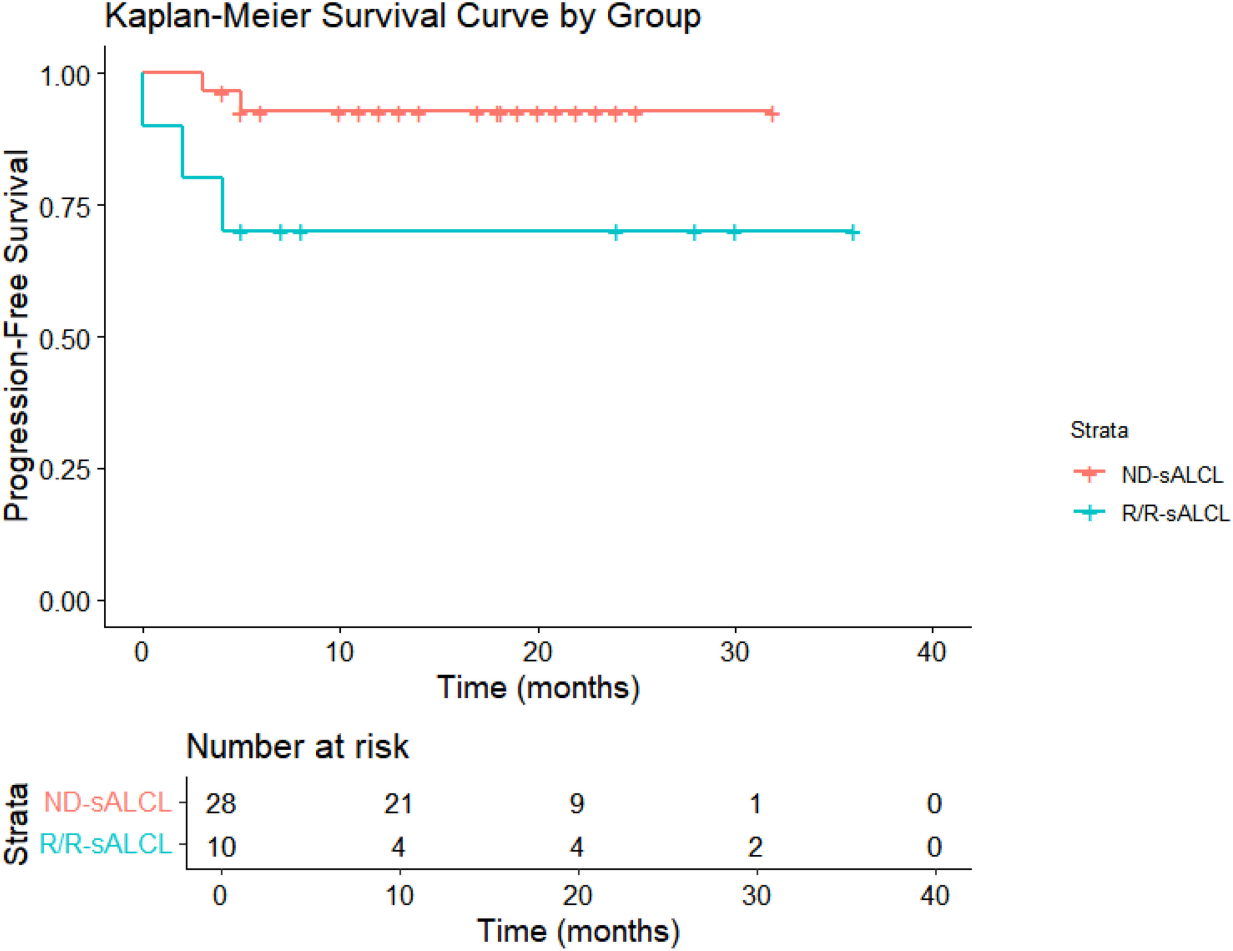
Progression free-survival.

**Figure 3:**
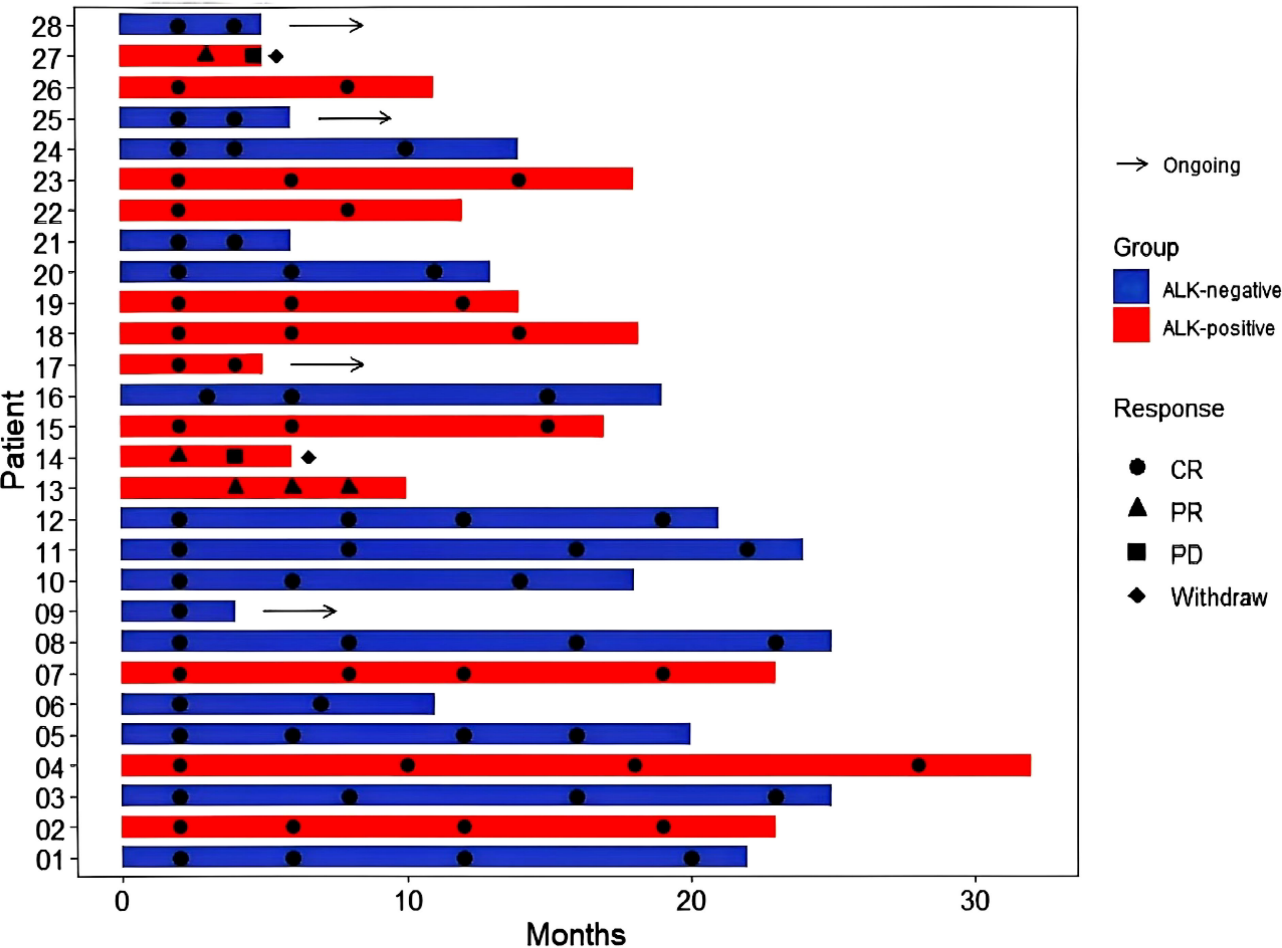
Response and survival status for ND-sALCL.

**Figure 4:**
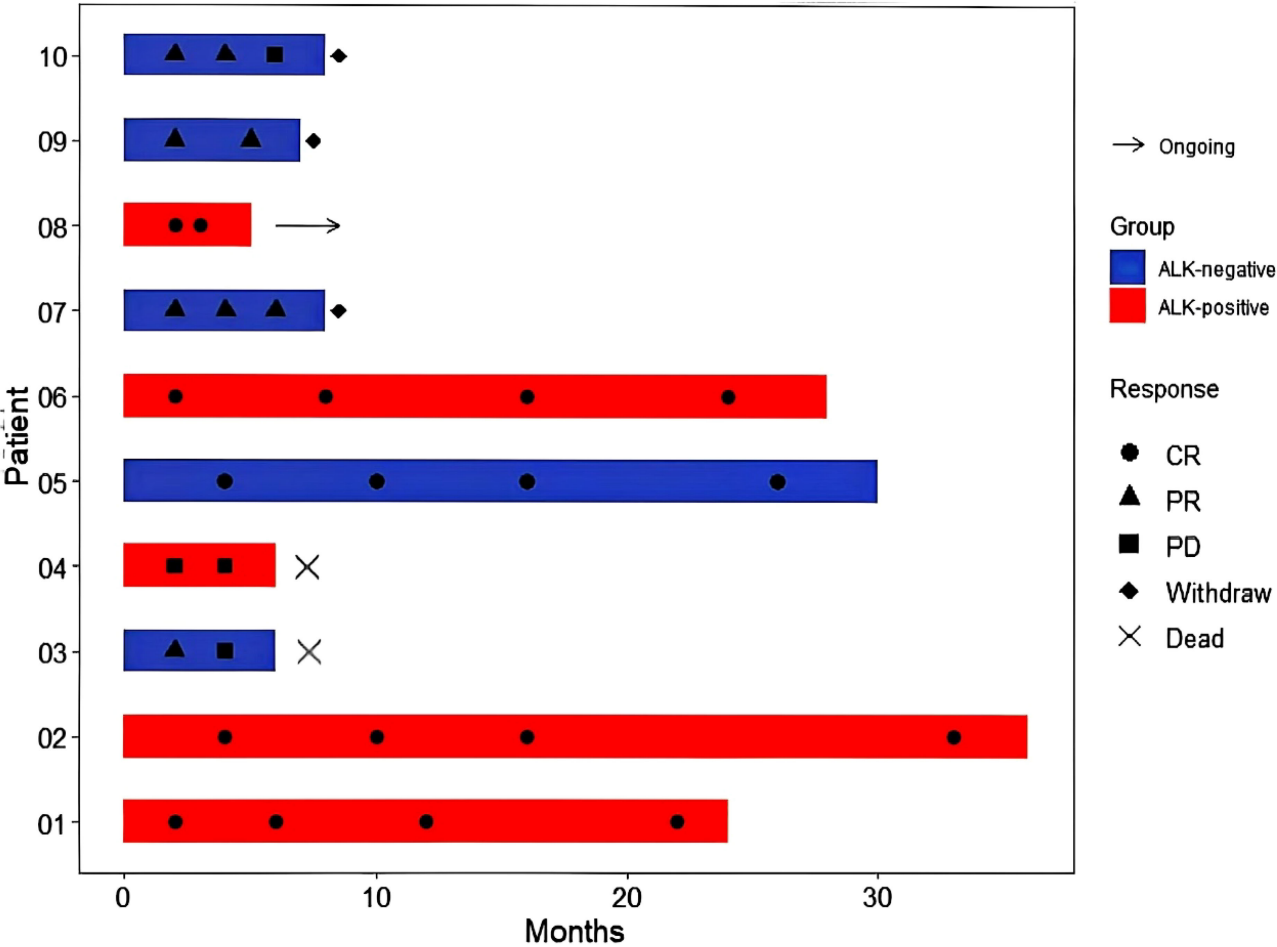
Response and survival status for R/R-sALCL.

Following a median observation time of 13.7 months(95% CI:12.5-18.3), The 1-year OS rates were 100% for ND-sALCL and 90% for R/R-sALCL. The 1-year PFS rates were 90% for ND-sALCL and 75% for R/R-sALCL, respectively. At the 2-year mark, the OS for ND-sALCL patients remained at 100%, while for those with R/R-sALCL it was 80% (**Figure 1**). The PFS was 92.8% for ND-sALCL patients and 70% for R/R-sALCL patients, with the median OS and PFS not yet reached (**Figure 2**). The clinical outcomes and response status are depicted for ND-sALCL (**Figure 3**) and R/R-sALCL (**Figure 4**).

### 3.4 Safety

All patients completed a minimum of four cycles of the BV combination regimen, with a median of 6.5 cycles administered (ranging from 4 to 8). The incidence of adverse events was 76.3%, The most commonly being peripheral sensory neuropathy (56.1%; grade≥3, 7.3%), neutropenia (31.7%; grade≥3, 22.0%), thrombocytopenia (26.8%; grade≥3, 12.2%) (**Table 3**). Overall, the treatment was well tolerated and had a toxicity profile consistent with previous reports.

**Table 3:** Incidence of AEs in the study.

AEs, n(%)	Total (n=41)	ND-sALCL (n=30)	R/R-sALCL (n=11)
Perpheral neuropathy			
Any Grade	23(56.1)	16(53.3)	7(63.6)
Grade≥3	3(7.3)	2(6.7)	1(9.1)
Leukopenia			
Any Grade	17(41.5)	9(30.0)	8(72.7)
Grade≥3	9(22.0)	4(13.3)	5(45.5)
Neutropenia			
Any Grade	13(31.7)	6(20.0)	7(63.6)
Grade≥3	9(22.0)	4(13.3)	5(45.5)
Anemia			
Any Grade	8(19.5)	3(10.0)	5(45.5)
Grade≥3	0	0	0
Thrombocytopenia			
Any Grade	11(26.8)	6(20.0)	5(45.5)
Grade≥3	5(12.2)	1(3.3)	4(36.4)
Infection			
Any Grade	8(19.5)	3(10.0)	5(45.5)
Grade≥3	2(4.9)	0	2(18.2)
Fatigue			
Any Grade	12(29.3)	8(26.7)	4(36.4)
Grade≥3	0	0	0
Vomiting			
Any Grade	9(22.0)	5(16.7)	3(27.3)
Grade≥3	0	0	0
Diarrhea			
Any Grade	3(7.3)	2(6.7)	1(9.1)
Grade≥3	0	0	0
Constipation			
Any Grade	8(19.5)	5(16.7)	3(27.3)
Grade≥3	0	0	0
Infusion reactions			
Any Grade	3(7.3)	3(10.0)	0
Grade≥3	0	0	0

AE adverse event, sALCL systemic anaplastic large cell lymphoma, ND-sALCL newly diagnosed sALCL, R/R-sALCL refractory/relapsed sALCL.

Peripheral neuropathy affected 23 out of 41 patients(56.1%),with 3 (7.3%) of patients experiencing grade≥3 peripheral neuropathy (**Table 3**). Notably, neurological toxicity generally resolved after treatment completion. Throughout the follow-up period, no long-term BV-associated toxicities were noted. The female patient undergoing BV retreatment having grade 2 peripheral neuropathy prior to restarting brentuximab vedotin also reported grade 2 peripheral neuropathy during treatment. It suggested that there is no superposition of neurotoxicity for BV retreatment.

In the study, the incidence of neutropenia was 31.7%,with 9 cases(22.0%)of grade≥3 neutropenia. The incidence of infection was 19.5%, which included 4.9% of patients with febrile neutropenia, and all occurred in relapse refractory sALCL patients.

The incidence of infusion reactions in the study was 7.3%, included fever (4.9%), chills (2.4%), and all occurred in the first time of BV infusion (**Table 3**). No pulmonary toxicity was observed and none of the patients discontinued treatment due to BV toxicity. There were no deaths occurred due to drug-related adverse events.

## 4 Discussion

BV has emerged as a pivotal sALCL treatment, demonstrating high overall remission rates in clinical trials for both newly diagnosed (4) and refractory/relapsed patients (5). However, real-world applications of BV-based therapies, especially in China, have been less well-documented, resulting in uncertainty about whether clinical trial results are applicable to everyday clinical practice. Our study bridged this gap by comprehensively analyzing BV’s efficacy in a real-world setting across multiple treatment centers, providing evidence of its effectiveness outside the stringent confines of clinical trials.

This observational, retrospective multi-center study involved 28 ND-sALCL patients treated with BV-CHP combination therapy, constituting the largest cohort reported on in a real-world setting to date. Our findings were broadly consistent with the ECHELON-2 study’s results. In the ECHELON-2 study, 70% of the participants were newly diagnosed with sALCL, and they found a 5-year PFS of 60.6% and a 5-year OS of 75.8% in the A+CHP group. Our study, with a short observation period, estimates a 1-year PFS rate of 95.0% and a 1-year OS rate of 100% in newly diagnosed cases. These findings suggest that the efficacy of BV, as observed in controlled settings, effectively translates to broader clinical practice. The ECHELON-2 study exclusively enrolled patients with III and IV disease and International Prognostic Index (IPI) scores greater than 2. In our cohort, 17.9% of the patients were in earlier disease stages, and 39.3% had IPI scores of less than 2 points. The proportion of ALK-positive patients was also higher in our study than in the ECHELON-2 study, which, along with the shorter follow-up period, likely contributed to the higher remission rates that we observed.

sALCL patients with multiple disease relapses are in a difficult position, with a dismal prognosis and treatment regimens that are focused on palliative care. The median OS for these patients is only about 3.0 months, and the median PFS is only about 1.8 months (9). Novel second-line treatment approaches that are less toxic but also effective are therefore needed for this patient population. Patients who could receive consolidative SCT would also need rapid disease remission (10, 11). Here, we retrospectively analyzed the efficacy and safety of BV combination regimens in 10 patients with refractory/relapsed sALCL. For these patients, the best ORR was 90% (with 50% CR), which dropped to 70% at the end of follow-up (with 50% CR). However, all of the CR patients maintained this status at the last follow-up, and the median response duration was 23.8 months. Here, we observe that the duration of CR in R/R sALCL patients is longer than that in newly treated patients. The primary reason for this disparity is that after BV was approved in China, its high cost and absence from medical insurance coverage initially led to its selection primarily for Refractory/Relapsed patients. Following the resolution of these issues, newly diagnosed patients started using BV. Extended follow-up periods may result in longer durations of complete remission (CR) for R/R patients. By the end of the follow-up period, the CR durations for four Refractory/Relapsed patients who chose BV-based combination regimens were 33.1, 25.8, 23.8, and 21.2 months, respectively. Coupled with the small number of cases, the results may not be reliable, and the analysis of large samples will be needed to draw exact conclusions. Two patients who were in PR at the time of their first response assessment progressed to PD during BV treatment. With a median follow-up time of 13.7 months, the estimated 1-year OS was 90% and the median had still not been reached. Similarly, the estimated 1-year PFS was 75%, and the median still had not been reached. The results were significantly better than those in the pre-BV literatures for R/R-sALCL patients. Our outcomes align with the prior pivotal phase II study, which reported an OS of 79% and a PFS of 57% at five years (5). Comparable efficacy has also been reported in an Italian study involving 40 patients, which demonstrated an ORR of 77.5% and a CR rate of 47.5% at the time of best response (12). Similar findings have also been observed in studies conducted within British and Korean populations (13, 14)**. **A notable difference between our study and the above studies pertains to the treatment regimen: we used a combination of BV and chemotherapy, and administration was capped at eight cycles. In contrast, they administered BV as a monotherapy every three weeks for up to 16 cycles. Until the present, most work on relapsed refractory sALCL have focused on single-dose BV treatment. Our study included the largest number of patients who were treated with BV combined with chemotherapy. Further clinical studies are needed to determine the merits of 6-8 cycles of BV combined chemotherapy versus 16 cycles of single dose BV.

Also we found several notable outcomes related to BV treatment of sALCL. Firstly, irrespective of whether the patients had ND-sALCL or R/R-sALCL, once a CR was achieved, its efficacy was sustained. Secondly, for patients only achieving PR after four cycles of BV combinations, the likelihood of transitioning to CR with additional cycles was virtually zero, suggesting that immediate treatment changes would be necessary for these patients. These findings mirrored those of a previous study in an Italian population (12).

For R/R-sALCL, second-line systemic therapy followed by consolidation stem cell transplantation (SCT) for those with a CR or PR is recommended in Clinical Practice Guideline. But the value of consolidation SCT in patients who have achieved a sustained response after BV treatment remains unclear. In our cohort, five ALK-negative ND-sALCL patients who reached CR underwent consolidative autologous hematopoietic stem cell transplantation. Conversely, nine ALK-negative patients with CR who did not receive SCT also had no disease progression at the time of the last follow-up. Among the R/R sALCL patients, three who achieved CR underwent consolidative autologous SCT, while two others who had completed eight cycles of consolidation therapy post-CR, one received radiotherapy, the other discontinue treatment, and they all remained in remission at the time of last follow-up. These findings suggest that a CR achieved with the BV-combined regimen forecasts a favorable prognosis regardless of further treatments, potentially negating the need for further SCT.

Reflecting on the pivotal phase II study, the five-year PFS rate was 68% for patients who underwent allogeneic SCT, compared to 47% for patients who did not receive a transplant after CR. This study found that 27.6% of their cohort achieved long-term remission exceeding five years with just single-agent BV (without other therapies, including stem cell transplantation). Another study reported a two-year disease-free survival rate of 54%, with 37.5% of patients in continuous CR and a median response duration of 12 months (range, 9-24 months). Only three of these 15 patients had undergone transplant consolidation (12), suggesting that long-term disease control is achievable without SCT. The durability of treatment responses and PFS in real-world settings indicate that patients with sALCL who achieve CR with BV monotherapy or combination regimen, no matter the newly diagnosed or the refractory/refractory patients, can attain prolonged disease control and may potentially be cured.

Nevertheless, for some refractory or relapsed patients, as well as patients with highly aggressive disease, stem cell transplantation may be required after multiple BV treatments. The available data suggest that BV does not adversely impact subsequent peripheral blood stem cell harvests, even among heavily pre-treated patients (15, 16).

The efficacy of continuing multi-cycle BV maintenance therapy after disease remission remains unclear. One study showed that BV could be active for MRD clearance and that multiple cycles of BV were necessary to achieve molecular CR in addition to clinical CR. dPCR quantification of MRD can be used to monitor treatment responses, and early positive MRD can be used as an indicator of relapse (17).

ALK-positive ALCL is generally associated with better clinical outcomes than ALK-negative ALCL. In our study, the ORR at the end of follow up for ALK-positive ND-sALCL was 92.3%, and 93.3% for ALK-negative ND-sALCL patients, and there is no significant differences between the two groups. However, among the 10 R/R-sALCL patients, four of the five ALK-positive patients achieved CR, and one had PR, while one of the ALK-negative patients achieved CR, two had PR, and two died. These findings suggest that ALK-negative patients with R/R sALCL may have worse treatment outcomes. No differences have been observed between ALK-positive and ALK-negative patients in previous clinical trials (6) or real-world studies (12). Thus, larger patient cohorts are required to substantiate these conclusions.

In the phase II clinical trial, long-term outcomes were not clearly associated with age (6). In a study by Broccoli et al. (12), the best response rate and ORR were among elderly patients. In our study, among the 28 ND- sALCL cases, five patients were older than 60 years old. Four of these patients achieved CR, and one patient achieved PR after two cycles of BV combined with CHP but then had disease progression after four cycles. Among the 10 R/R-sALCL patients, there were two elderly patients over 60 years old. One of these patients was 78 years old and achieved CR for 30 months, and the other patient achieved PR. Our small sample size precludes statistical analysis but does imply that BV’s efficacy in elderly patients may be comparable to its efficacy in younger individuals.

In our study, one female patient who had been previously treated with BV combined with CHP as a first-line regimen and achieved CR then relapsed nine months after treatment was discontinued. She suffered from bone marrow invasion and a large neck mass before restarting BV with ICE. Her best response was PR, and her disease progressed six months later, eventually leading to mortality. The safety and antitumor activity of BV retreatment has been investigated in a study involving patients who had previously achieved CR or PR with BV. This prospective study reported favorable efficacy, with an ORR of 88% (63% CR) in sALCL (18). Another retrospective, multi-center observational study assessed the safety and efficacy of BV retreatment in patients with sALCL, and found an ORR of 70% (60% CR). The safety profile was generally similar to that seen in initial BV treatment (19). These authors suggested that the decision about whether second-line BV retherapy should be used after first-line BV treatment should take the interval of relapse and CD30 expression density into consideration. One study analyzed loss or decreases in CD30 expression in four patients with ALCL after BV-containing therapy. They found that 44% of ALCL patients, regardless of histological subtype, had a complete loss or decrease in CD30 expression after BV-containing therapy (20).

In comparison to the toxicity profiles demonstrated in clinical trials, we found no additional safety signals, even when BV was used in combination with conventional agents.BV had a favorable toxicity profile overall. Adverse effects were primarily neurological and were seldom severe enough to necessitate treatment reduction or interruption, with no overlapping toxicities observed. Neurological toxicities are typically fully resolved post-treatment. No other long-term toxicities were found during the follow-up period. BV is also well-tolerated in pediatric populations (21, 22). However, it is important to note that, in a study evaluating the safety profile of BV in Japanese patients, the incidence of pulmonary toxicity was 4.6% (grade ≥ 3, 3.9%) in the overall population, including interstitial lung disease in 3.9% of patients, respiratory failure in 0.7%, and acute respiratory distress syndrome in 0.4% (23). Although these events are rare, they are also potentially fatal, so close monitoring may be warranted, especially in patients with either a history of or ongoing pulmonary disorders.

In conclusion, our multi-center retrospective study provides compelling evidence that BV is an effective and well-tolerated treatment option for both newly diagnosed and refractory/relapsed sALCL. The rapid induction of clinical responses and the maintenance of efficacy, combined with its favorable safety profile, suggest that BV could be a valuable addition to the therapeutic landscape of sALCL in China. While acknowledging the limitations inherent to the study design, the real-world data presented here reinforce BV’s potential to improve clinical outcomes in sALCL. It is imperative to continue research efforts that can further clarify BV’s treatment role and limitations and improve the lives of patients battling this challenging malignancy.

## Data Availability

The raw data supporting the conclusions of this article will be made available by the authors, without undue reservation.

## Supplementary material

Supplementary Table 1 Treatment history of R/R patients.
