# Supplementary material for: Real-world experiences with brentuximab vedotion-based regimens in systemic anaplastic large cell lymphoma: a multi-center retrospective study
Source: Front Oncol. 2025 Jan 7;14:1494384. doi: 10.3389/fonc.2024.1494384 (PMC11746031; doi:10.3389/fonc.2024.1494384)
Supplement: Supplementary Table 1 — Treatment history of R/R patients. [file Table1.docx]

| Patient | First line | Second line | Third line | Fourth line | Fifth line | BV-based regimen |
| --- | --- | --- | --- | --- | --- | --- |
| Case 1 | CHOEP-ASCT |  |  |  |  | BV+ICE |
| Case 2 | CHOP |  |  |  |  | BV+GDP |
| Case 3 | BV-CHP |  |  |  |  | BV+ICE |
| Case 4 | CHOP-ASCT | GDP |  |  |  | BV+ICE |
| Case 5 | COEP | ICE | PI3K Inhibitor |  |  | BV+GeMox |
| Case 6 | CHOP | ICE | Mitoxantrone liposomes |  |  | BV+GDP |
| Case 7 | CHOEP | ICE | Mitoxantrone liposomes |  |  | BV+GDP |
| Case 8 | CHOEP | GeMox | Mitoxantrone liposomes | PI3K Inhibitor |  | BV+ICE |
| Case 9 | CHOP | ICE | Mitoxantrone liposomes | PI3K Inhibitor |  | BV+GeMox |
| Case 10 | CHOP | ICE | chidamide | Mitoxantrone liposomes | PI3K Inhibitor | BV+GDP |

Suppliment Table1. Treatment history of R/R patients.
